# Trainable movement control using spikes and muscle-twitch dynamics

**DOI:** 10.3389/fnbot.2026.1761767

**Published:** 2026-04-13

**Authors:** Jordi Timmermans, Lambert Schomaker

**Affiliations:** 1 Department of Artificial Intelligence, Bernoulli Institute for Mathematics, Computer Science and Artificial Intelligence, University of Groningen, Groningen, Netherlands; 2 Groningen Cognitive Systems and Materials Center (CogniGron), University of Groningen, Groningen, Netherlands

**Keywords:** ballistic control, direct feedback alignment, global error, neuromorphic, neuromuscular inspiration, Pong, spiking neural networks, twitch

## Abstract

The biological sensorimotor system is a source of inspiration for the design of neuromorphic ballistic control systems. A large portion of sensorimotor-inspired research focuses on the sensory encoding and information processing stages of the system. However, research on broader task-performance systems, involving actuator control on the output side remains scarce. In this work, we develop and train a neuromuscular-inspired model to perform ballistic control. In the model, a spiking neural network's output spikes are used to generate twitch-like signals. These twitches are the basis for generating a continuous fluctuating output signal that is used to operate an actuator. We refer to the the used model as the Twitch Neural Network (TwNN). As a test case, the model is trained to control the paddle of an adapted version of the game of Pong. An adapted version of the Direct Feedback Alignment learning rule, specifically for integrate-and-fire neurons, is introduced. The new rule avoids the update-locking problem of backpropagation, allowing network weight updates in parallel. The model output consists of one group of agonist-innervating motor neurons, and one group of antagonist-innervating motor neurons. We find that it is possible to teach a neuromuscular-inspired system to control the paddle in the game of Pong with the adapted Direct Feedback Alignment learning rule. The best-performing baseline model achieved a hit rate of 96%. By applying logarithmic scaling to the output activity, a hit rate of 98% could be achieved. Finally, by replacing the neuromorphically unrealistic exact summation steps with leaky integrators in training, the range of good learning parameters became more narrow and clear. The best-performing model reaches a hit rate of 99%. Threshold analysis during training has shown that learning is robust to a variety of neuron thresholds. Noise analysis has shown that the system is robust to membrane potential noise during inference for uniform noise up to values in the order of around 0.1-1% of the neuron threshold value per time step.

## 1 Introduction

Spiking neural networks (SNNs) (Maass, 1997; Gerstner and Kistler, 2002) have shown to be effective information processors. These network models are closer to biological neural systems and their performance will be similar to that of traditional continuous floating point-valued neural networks (Rueckauer et al., 2017). More importantly, SNNs provide attractive advantages for implementation in more energy-efficient electronic (neuromorphic) hardware (Davies et al., 2018). This energy-efficiency is possible due to the fact that in individual processing elements are in a resting state for most of the time, unlike continuously clocked digital electronics that is currently used for information processing. For edge computing and analog control, there are interesting challenges both at the input and the output. On the input side, the processing changes from continuous value evaluation to an event-based paradigm. Several solutions are being proposed in literature for different sensory modalities ranging from touch (Mastella and Chicca, 2021) to vision (Richter et al., 2023) and audio (Baek and Lee, 2024). On the output side, less research has been done on optimized analog control by these spiking models. The challenges are in adapting the abrupt, discrete nature of spiking signals to the smooth analog patterns that are needed for the control of physical systems. In earlier work, we took inspiration from the biological, neuromuscular control systems. Rather than utilizing the spikes directly as an output control signal, or use overly simplistic low-pass filtering, we proposed to use the muscular twitch. A twitch is a second-order damped impulse response, as a natural output stage for a spiking neural controller (Schomaker et al., 2023). This mechanism is vastly present in the animal world, and provides survival capabilities in virtually all animal species (Nishikawa et al., 2007). In our previous work, we taught a twitch-based system to control the inverted pendulum. In this work, we adapt the system to tackle more complex tasks. Most importantly, where previously only single twitch output timings and time-constants were trainable, we now train a spiking neural network to produce muscle-twitch bursts. These bursts allow for more flexible control, as twitch frequency and time can be adapted within each burst. The adaptation of output spike to muscle twitch in a neuromorphic setting does require new learning rules, which is the main focus of this work.

When an output spike train itself would be used for control, it would give rise to a jittering movement. A way to reduce the spike-induced jitter is to low-pass filter the output spikes. By using a (weighted) average over the last N spikes, a spiking neural network is used to turn a robot (Wang et al., 2008) or to perform a lane-following task (Kaiser et al., 2016). Simple moving average filters however, require precisely tuned time windows, and they also require saving historical data. A good alternative way to smoothen the output spikes to approximate a movement pattern is to applying a convolution operation with a smoothing filter to these spikes. A suitable smoothing filter is the Gaussian filter, as the bell shape can be used as a universal density function approximator (Goodfellow et al., 2016). This wave shape however, is not a natural impulse response of physical systems: In a realizable causal system, the left-hand tail cannot start at *t* = −∞, and a (truncated) bell shape would be rather expensive to produce for each output neuron in an electronic circuit. From the biological neuromuscular system, we can observe that the interfacing between neuron and muscle, which together form the motor-unit, allows for more smooth control. The output spikes of the motor neurons activate multiple muscle-fibers, which contract with a second-order overdamped impulse response (Liddell and Sherrington, 1925; Duchateau and Enoka, 2011). The distribution of time constants in the system allows for both fast and slow twitches (Henneman et al., 1965; Fuglevand et al., 1993). The cheaper and more biologically plausible alternative would thus be to use second-order overdamped impulse responses, the impulses being the output spikes, as a spiking neural network output for control. Similar to the human neuromuscular system, an ensemble of filtered outputs can perform the desired movement pattern. For neuromorphic computing purposes, the filter can also be realized in electronics, using two cascaded RC filters. In an earlier published article, we have shown that the principle of using an ensemble of motor-unit twitches is usable in, e.g., an inverted pendulum task (Schomaker et al., 2023). However, timing in the inverted pendulum task is partly governed by explicit constraints. In other tasks, which involve time-varying sensory inputs and variable output timing requirements, a learning SNN would need to learn effective timing patterns for output twitches itself.

**Figure 1:**
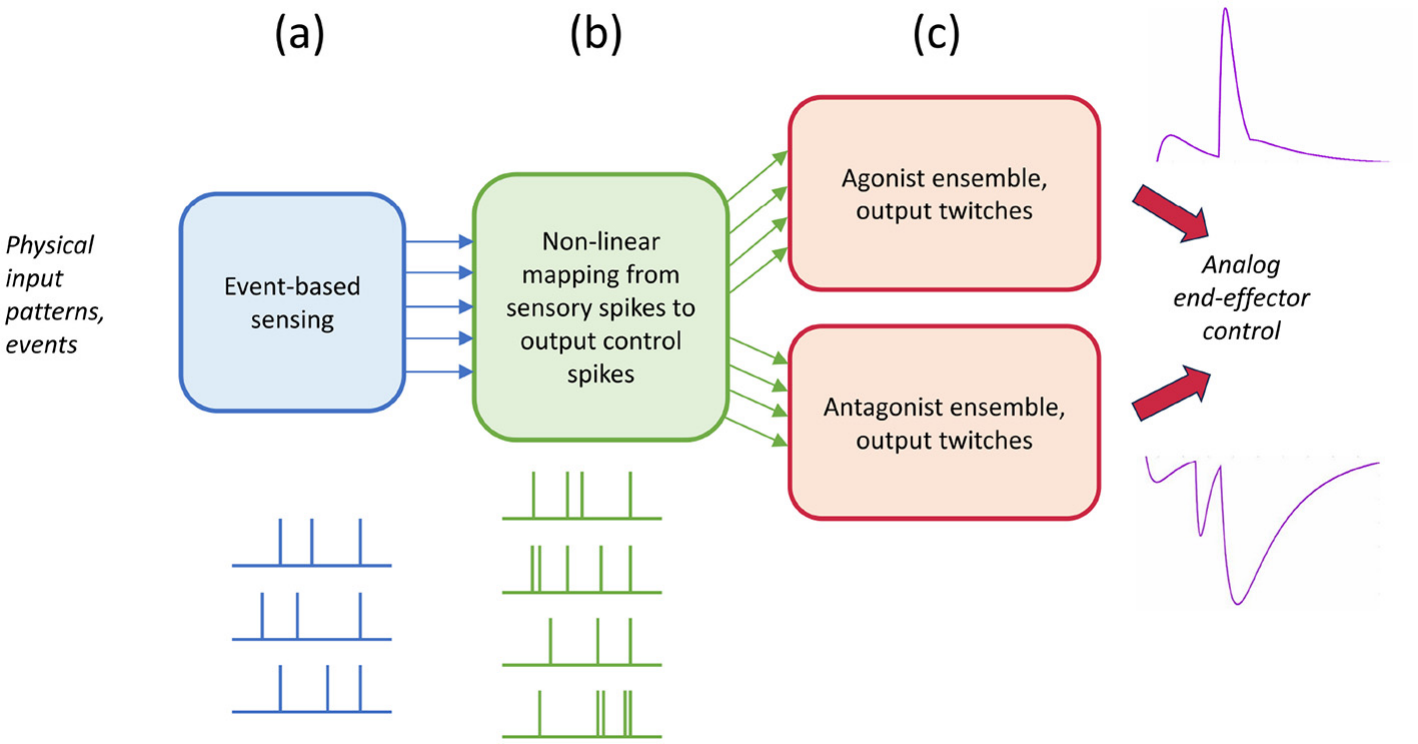
Overview of the general spikes & twitches control approach in three stages: **(a)** Event-based sensing, **(b)** non-linear mapping and **(c)** an ensemble of twitch generators at the output (right), directed toward an agonist and antagonist pool to deal with bipolar control requirements.

Spikes and muscle twitches represent only positive signals. Therefore, a problem arises when an actuator needs to be able to move in two opposing directions. In anatomy we can see this in, for example, the elbow, where a joint can only rotate on one axis (1DoF). This requires an agonist which pulls the joint angle in one direction and an antagonist, which pulls the joint angle to the opposite direction. In case of a simple simulation of 1 DoF neuromuscular systems using an SNN, this means that one set of neurons should be assigned to the agonist muscle, and another set of neurons should be assigned to the antagonist muscle. These two opposing actuators avoid the use of impractical negative signals on the neuron output in an electronic implementation. In addition, the use of antagonistic outputs avoids the use of inverter circuits. Figure 1 shows a diagram of the general approach. In this work, the event-based sensing system (Figure 1a) is given, but adaptivity, i.e., learning will be needed in the non-linear mapping of sensor toward motor patterns and the twitch-generation output stage (Figures 1b, c).

There are many types of control tasks. In this study, we focus on object interception. Two theories dominate the object interception task: prospective and predictive control. In predictive-control theory, perceptual information is gathered while viewing the object. When enough information is perceived, the muscles are triggered to make the correct movement. In prospective control there is continuous feedback while trying to intercept the object. We focus on predictive control theory, specifically ballistic control. In this type of control, information is gathered, after which an all-out, barely-adjustable effort is produced to make an accurate goal-directed movement. Fast ballistic control is needed in some actions because closed-loop control may be hampered by neural and neuromechanical delays. Examples of this type of control in animals are the *archerfish* (Schuster, 2018; Gerullis and Schuster, 2014) that shoot their prey with a water jet, and *jumping spiders* (Jackson and Pollard, 1996) that jump toward their prey. *Humans* use this type of control mainly in striking, kicking, hitting and throwing (Stark, 1982; Elliott et al., 2010). Elliott et al. (2010) also makes the point that after the parameterization of the ballistic movement, control commands can be sent downstream, freeing resources for the planning of a next action.

The neuromuscular system continuously adapts to new tasks and environments. Teaching a neuromorphically plausible neuromuscular-inspired model to perform tasks is a challenge. Despite the great success of backpropagation (Rumelhart et al., 1986a) and gradient descent in digital feedforward neural networks, neuromorphic computing generally requires different algorithms to learn. Neuromorphic algorithms need to learn locally, preferably without the need to monitor their own weight values. STDP would be a straightforward choice, as this rule only uses information in the pre- and post-synaptic neurons to learn. The STDP rule however, is only able to find correlation between neurons in a basically Hebbian manner and cannot differentiate and utilize the occurrence of good or bad action outcomes.

Reward-modulated STDP: Reward-modulated STDP (Izhikevich, 2007) can be a solution for this differentiation. This rule modulates the weight updates as a result of leaky-integrator-based spike-timing information and an additional general reward signal for the current action instance. When using R-STDP, it is important to parametrize the leaky integrator such that the reward at the end of a trial can be evaluated properly in spite of vanishing spike-time information. A relatively late appearance of reward information is typical for ballistic control tasks. However, there exists a more serious limitation of R-STDP. In a neuromuscular system, output spikes are connected to motor units. Each motor unit implicitly weighs the effect of its output spike through its individual force effect on the total final output. It is not self-evident how a general single (scalar) error signal, as used in R-STDP, can utilize the individual force effects to aid training. Feedback-alignment: A second option for learning are the feedback-alignment based algorithms. The authors of the feedback alignment (FA) algorithm (Lillicrap et al., 2016) discovered that it is not necessary to have symmetrical feedforward and feedback weights for a neural network to learn. Instead, a static, randomly initialized feedback matrix is constructed, called *B*. Surprisingly, *B* becomes aligned with the transpose of the feedforward weights *W*^*T*^ in the iterative training process. This allows useful error information to flow back into *W*. The alignment can be sufficient for learning to take place, if the resulting gradient deviates not more than 90° from what back propagation would specify. An initial settling stage puts the feedback process on track. FA avoids the need for storing and retrieving the weight values, as it feeds back the learning signal through a random and non-plastic feedback matrix. In contrast to R-STDP, this learning rule is able to utilize individual force effects for training. It can scale each output neuron's contribution to the general output with the scalar general error. As such, an error vector can be created, assigning an individual error to each output neuron. The error vector, feedback matrix and input to the weight matrix can be used to update the weight matrix for the output layer. Consequently, information that includes muscle information can flow back into the network.

Direct feedback-alignment: One issue to be solved is that FA still requires storing the gradient vector for each layer. This is time-inefficient since weight updates are locked until the previous layer's gradients are calculated. A learning rule that prevents such update locking while still using the FA principle is the Direct Feedback Alignment (DFA) rule (Nøkland, 2016). Compared to feedback-alignment, DFA feeds the error vector directly rather than sequentially into each weight matrix through a random feedback matrix. The result is an approximation of the local gradient and is multiplied with input activity from the previous layer to update the weight values. While DFA does not get rid of layer-based weight locking as a consequence of having to wait for the forward pass to complete, it does allow for updating all weights simultaneously, using only a global error signal.

### 1.1 Research questions

Spiking neural networks have shown to be usable in control systems. Yet, spike trains are usually decoded to generate movement. Adding a muscular-inspired output can be used to generate smooth movements, on the basis of temporal information. Given this premise, we asked ourselves the question: Is it possible to train a neuromuscular-inspired spiking neural network, i.e., an SNN with second-order overdamped impulse responses as output stage to perform a control task? Similar research has been performed in (Kang and Banerjee, 2017), and (Huebotter et al., 2025). Although they both use backpropagation, which is not neuromorphically plausible.

Several requirements had to be met in order to make the system neuromorphically plausible:

- No update locking results from the learning process as in backpropagation and feedback alignment
- There is a simple way to use the task performance error during training
- The system must be usable on memristive networks.
- In anticipation of ultimate circuit implementations, the complexity and dependencies need to be limited

**Figure 2:**
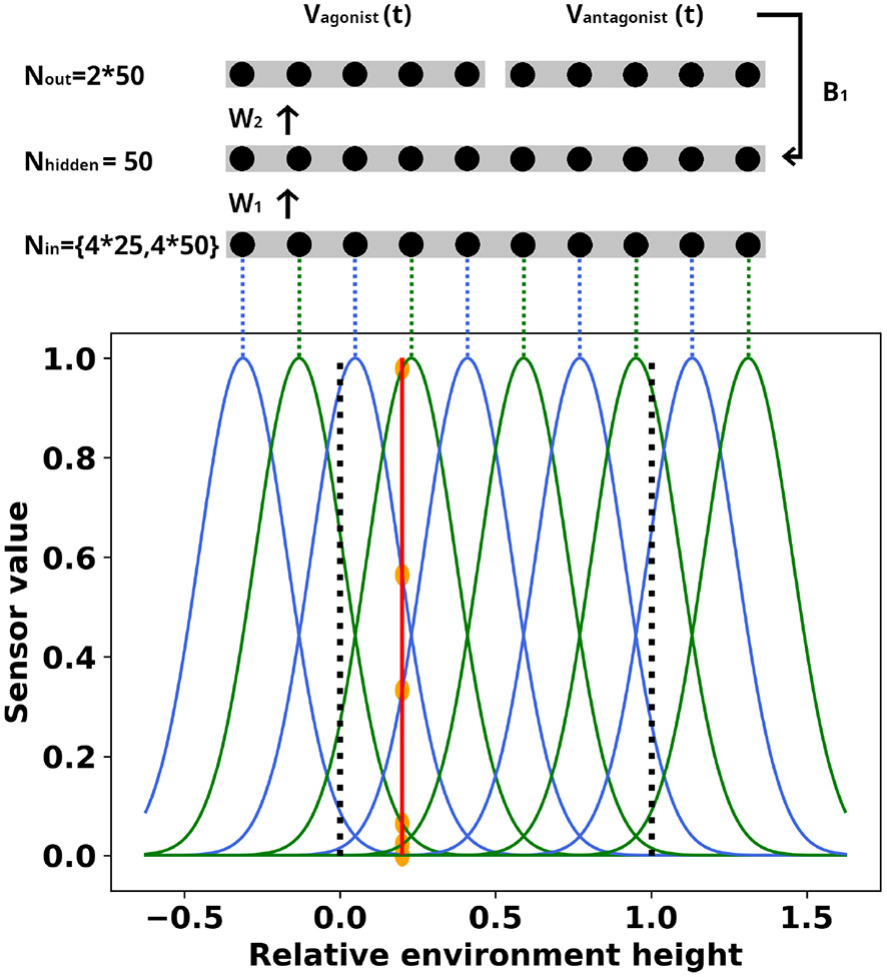
Gaussian encoding of the perception of the environment from the viewpoint of a single simulated ToF sensor, connected to the neural network. Note that the input consists of four concatenated groups of neurons, one for each sensor. The example shows the encoding of the ball position into neural activity for a single sensor. The positions of the horizontal environment walls are indicated with the black dotted line. An example of sensor value conversion of a ball crossing the sensor's receptive field is indicated by the red solid line and the orange discs. The red solid line indicates the crossing position, while the orange discs indicate the used sensor value per neuron. The feed-forward spiking neural network will generate a spiking output, with an implicit output function. This output function is used to move the paddle. Network weights are updating according to Direct Feedback Alignment, using a random matrix B.

We propose a neuromorphically-plausible trainable neuromuscular-inspired model that is able to learn to perform control tasks for intercepting moving objects, using ballistic movements. Please note that we do not intend to exactly model the neuromuscular system but only take inspiration from it. The system encodes sensor information using overlapping Gaussian receptive fields. The encoded value will be used to stochastically produce events that cause the input neuron to emit a spike. The spiking neurons in the following layers are made up of integrate and fire (IF) neurons. Each IF neuron in the output layer is connected to a single simulated motor unit. As a result, rather than relying on counting spikes in a predefined time frame to decide what action to take, the action is continuous and inherently connected to the spikes generated by the output neurons. See Figure 2 for an overview of the system. We also propose a novel approach to update the spiking neural network's weights. We introduce a spiking version of the DFA learning rule, specifically for integrate and fire neurons. This learning rule avoids the need to read out weight values, or store them in FPGA controllers. We call it Spiking Integrate and Fire Direct Feedback Alignment (SIF-DFA). We test the system on an adapted version of the game of *Pong* (Atari, 1972), the adapted version restricts the system to predictive control.

### 1.2 Contributions

The contributions of this paper are as follows: we provide an extension of our earlier proposed model (Schomaker et al., 2023) using timed neuromuscular twitches in the output stage for an analog controller. The full model, referred to as the Twitch Neural Network (TwNN), is a spiking neural nework in which each output neuron spike produces a muscle-twitch like signal. We extend the list of tasks (inverted pendulum and handwriting trajectories) with the task of playing the game of Pong. This task has more requirements at the level of perception and spatiotemporal actuation, especially regarding predictive capabilities. For this task, it was necessary to use a three-layer TwNN. This also required extensive adaptation to the learning rule and the learning process. Contrary to our previous work, the current model also allows for spike bursts per output unit. After pilot experiments and a parameter sweep, the best basic model reached a hit rate of 96%. The system's performance was improved by taking the log scale of the error signal. After this scaling, the best model reached a hit rate of 98%. The tested systems utilized the exact summation of spikes and neural activity to calculate the weight update. By replacing the summation by an electronically more viable leaky integrator, the performance of the good parameter combinations increased, the best-performing model reaching a 99% hit rate. With the latter modification, the system will also be easier to implement in electronics. Additionally, we have performed analysis of threshold variations in learning, and we performed membrane potential noise analysis during inference. The model training code, the code to generate plots and some additional visualization tools are made available on GitHub.^1^

## 2 Methods

The pipeline of training the proposed neuromuscular system consists of four stages:

1. Sensor information is measured and encoded such that it can be used by a spiking neural network (Section 2.1);
2. The spiking part of the neural network processes the sensor reading to determine optimal timing and amplitudes (Section 2.2);
3. Output spikes are translated into movement by means of a muscular-inspired final actuation output (Section 2.3);
4. A reward signal is calculated and fed back in the network to tune the weights (Section 2.4).

A description of the design choices for the Pong environment will be presented (Section 2.5).

### 2.1 Measurement and encoding of ball positions

Residing outside of the neuromorphic model, the sensor input to the network model is assumed to be a given constraint, to which the TwNN needs to adapt itself. However, the chosen sensor and perception model is designed to be compatible with neuromorphic processing in the next stage in the pipeline. Simplified models of Time of Flight (ToF) sensors measure the distance of an object to the sensors, when the object passes through the laser. The sensors need to be strategically placed, such that there is enough information to take the appropriate action. In our system, sensory signals are encoded using a distributed representation (Rumelhart et al., 1986b), i.e., a representation where each sensory event is encoded by multiple input neurons, and each input can partake in encoding a variety of events. Each ToF-like sensor output is connected to a group of input neurons, as can be seen in Figure 2. Each neuron in this group has a Gaussian receptive field on the ToF-sensor. Note that while Gaussian functions in time are impractical to generate, Gaussian-like voltage-to-current mapping circuits exist and are termed *bump circuits* (Delbrueck and Mead, 1993; Moustakas et al., 2025). The means of the Gaussian receptive fields are equally spaced, and the variances are prespecified. Some of the Gaussian means lie outside of the environment. This realizes homogeneous sensor-value conversions over the whole environment, preventing ToF sensor crossings near the boundaries of the environment to significantly activate a smaller number of neurons than is the case in the more centrally concentrated readings. For generalizability, the ToF-sensor's output value is scaled linearly between 0 and 1. Each Gaussian receptive field maps the normalized ToF-sensor onto a value *h* that is used to determine the corresponding input neuron's spike rate. We represent our measured values similarly to (Bohte et al., 2002), but instead of mapping a value to spike timings, we map the values to frequency.

### 2.2 Information processing and actuation

Gaussian-encoded information is processed by an artificial spiking neural network. The input layer of the network consists of neurons that fire probabilistically. The probability of a neuron emitting a spike *p*(*s*) follows a Poisson process that is expressed in Equation 1, where *s*(*t*) is a spike on time *t*, *c* controls the width of the Gaussian receptive field, and *x*−*b* represents the distance of the measured object to the mean of the Gaussian. Parameter ζ represents the excitability of the input neuron, and is a tunable parameter.


$$\begin{array}{l} p\left(s\left(t\right)\right)=\frac{1}{\zeta }\;e^{-c{\left(x-b\right)}^{2}}=\frac{h}{\zeta } \end{array}$$ (1)

Timesteps Δ*t* = 1 *a*.*u*., the average spike rate will be ν = *p*(*s*(*t*)).

The spiking neural network consists of fully-connected layers with trainable network weights *W*_*l*_. Each presynaptic neuron's output spike is scaled using a synaptic weight *w*. Each post-synaptic neuron receives and accumulates weighted spikes. The sum of received weighted spikes is added to the neuron's state variable *u*, akin to the membrane potential. The state variable update can be seen in in Equation 2.


$$\begin{array}{l} u\left(t+1\right)=u\left(t\right)+{\text{s}}_{pre}\left(t\right)\cdot \text{w}+R\left(t\right) \end{array}$$ (2)

If the membrane potential crosses the neuron's spiking threshold θ, the reset mechanism *R* is activated. During the reset, the membrane returns to it's resting potential of *u* = 0 and emits an output spike *S*(*t*). The discharge process can be seen in Equation 3.


$$\begin{array}{l} s_{out}(t)=\{\begin{array}{cc} 1, & \text{if }u(t)\geq \theta \\ 0, & \text{if }u(t)<\theta \end{array} \end{array}$$ (3)

### 2.3 Actuation

**Figure 3:**
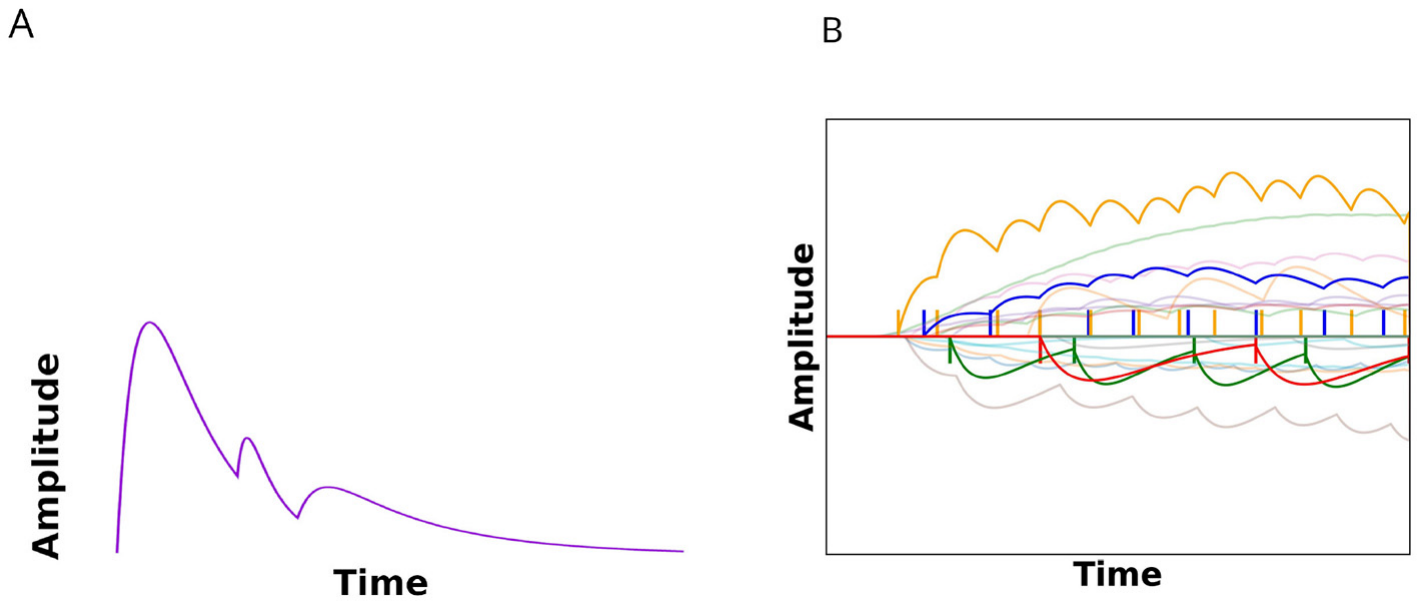
**(A)** Summation of three twitches from three different output neurons. Each twitch is parameterized using an amplitude and two time constants. **(B)** Example of one single trial with 50 agonist (upper half of the image), and 50 antagonist (lower half of the image) muscle outputs. Note that both signals are positive, but change the velocity of the actuator in opposite directions. For clarity, the antagonist's twitches are drawn as negative signals. A summation of signals from the a(nta)gonist twitches results in the net amplitude of the respective muscular signal. The difference in net amplitude at each timestep corresponds to the velocity of the actuator. For each muscle, two motor neurons and corresponding motor unit twitches are highlighted.

Similar to the biological system, where a motor unit is innervated by one motor neuron, each output neuron in our system has a one-on-one connection with an amplified second-order impulse response filter. Every filter is parameterized by two randomly sampled time-constants and a randomly sampled output amplitude. The filter parameters are static and cannot be trained. A simple summation of different time-constant and amplitude twitches can be seen in Figure 3A. Analogous to biological antagonistic actuator pairs, our system also consists of one set of agonistic motor units, and one set of antagonistic motor units. This gives control of one set of output neurons to one movement direction, while the other set has control over the opposite direction. Antagonistic control allows actuation solely with positive output signals. An example of a control trial can be seen in Figure 3B, the antagonist is shown on the negative y-axis. The conversion from spike to actuation is described by Equations 4–6.


$$\begin{array}{l} c_{1}\left(t\right)=c_{1}\left(t-1\right)\;\alpha +\left(1-\alpha \right)\;s\left(t\right)\;A \end{array}$$ (4)

$$\begin{array}{l} c_{2}\left(t\right)=c_{2}\left(t-1\right)\;\beta \;\left(1-\beta \right)\;c_{1}\left(t\right) \end{array}$$ (5)

$$\begin{array}{l} v\left(t\right)=c_{2}\left(t\right) \end{array}$$ (6)

*c*_1_ and *c*_2_ are the filter's state variables, α and β are the filter's time constants where $\alpha =exp\left(\frac{-1}{\tau_{rise}}\right)$ and $\beta =exp\left(\frac{-1}{\tau_{fall}}\right)$. Contrary to nature's muscles which generate force while contracting, our outputs are velocity *v*(*t*) controllers. The state value of the motor unit equals the velocity it contributes to the momentary velocity of the actuator. The cumulative state values of the *N* motor units is equal to the net velocity of the actuator (Equation 7).


$$\begin{array}{l} {\text{v}}_{net}\left(t\right)=\overset{N}{\underset{i=1}{\sum }}{\text{v}}_{i}\left(t\right) \end{array}$$ (7)

### 2.4 Learning using a scalar global error signal

In a fully connected neural network of three layers, the forward pass is described by Equations 8–9. Inputs **x** or **h**_*n*_ are multiplied by their respective weight matrix *W*_*n*_. The output vectors **a**_*n*_ are transformed through an activation function *f*, resulting in activation vectors **h**_*n*_


$$\begin{array}{l} {\text{h}}_{1}=f\left({\text{a}}_{1}\right)\;,\;{\text{a}}_{1}=W_{1}\text{x} \end{array}$$ (8)

$$\begin{array}{l} {\text{h}}_{2}=f\left({\text{a}}_{2}\right)\;,\;{\text{a}}_{2}=W_{2}{\text{h}}_{1} \end{array}$$ (9)

DFA updates network weights by multiplying the error signal, using a random feedback matrix *B*, an error vector **e**, the local derivative of the activation *f*′(**a**), and the input to the weights **x** or **h**. The output layer's weights can simply be updated by only the error vector and the input to the weights. Weight updates for such network are performed using Equations 10–12. “⊙” represents the Hadamard product.


$$\begin{array}{l} \delta W_{1}=-\delta {\text{a}}_{1}{\text{x}}^{T}\;,\;\delta {\text{a}}_{1}=\left(B_{1}\text{e}\right)⊙f^{\prime }\left({\text{a}}_{1}\right) \end{array}$$ (10)

$$\begin{array}{l} \delta W_{2}=-\delta {\text{a}}_{2}{\text{h}}_{1}^{T}\;,\;\delta {\text{a}}_{2}=\left(B_{2}\text{e}\right)⊙f^{\prime }\left({\text{a}}_{2}\right) \end{array}$$ (11)

$$\begin{array}{l} \delta W_{3}=-\text{e}{\text{h}}_{2}^{T} \end{array}$$ (12)

DFA is originally made for feedforward neural networks. Conversion of the rules to rules usable for our spiking neural network requires adaptations in **x** and **a**_*n*_, such that information over multiple timesteps can be represented in those variables. This also means that, in electronics, we would need to have a parallel circuit per neuron, that keeps track of its history. We name our learning rule Spiking Integrate and Fire - Direct Feedback Alignment (SIF-DFA). In SIF-DFA, we replaced *x* by the expected input spike rate ν, which we can directly obtain as shown in Equation 1, given Δ*t* = 1. The activation values **a**_*n*_ over one trial are obtained by summing all weighted spikes, i.e., ${\text{a}}_{1}=\overset{T}{\underset{0}{\sum }}W_{1}\text{x}\left(t\right)$ and ${\text{a}}_{2}=\overset{T}{\underset{0}{\sum }}W_{2}{\text{h}}_{1}\left(t\right)$. For calculating the activations, we chose *f* to be the sigmoidal function, parametrized by steepness *s*, as shown in Equation 13.


$$\begin{array}{l} f\left(x\right)=\frac{1}{1+e^{-x/s}} \end{array}$$ (13)

Weight updates can be performed either per trial or after multiple trials. When updating after multiple trials, usually, the update directions are saved in a buffer and averaged after a number of trials. In order to avoid writing additional values to buffers, we chose to provide feedback using a single trial. After a trial has ended and the result is acquired, weight updating is initialized. We first calculate the goodness of the attempt *E* = *y*_*b*_−*y*_*p*_, the distance between the ball *y*_*b*_ and the middle of the paddle *y*_*p*_. We also calculate an estimate for the individual contribution per output neuron *c*_n_*A_*n*_, where $c_{n}=\overset{T}{\underset{0}{\sum }}s_{out}\left(t\right)$ is the sum of output spikes of the neuron over one trial and *A*_*n*_ is the amplitude of the filter innervated by the neuron. The calculation of the individual error of a motor neuron is shown in Equation 14.


$$\begin{array}{l} e=Ec_{n}A_{n} \end{array}$$ (14)

The feedback error vector is composed of all individual errors: $\text{e}=\left(\begin{array}{c} e_{0}\\ ...\\ e_{n} \end{array}\right)$

Note that although exact spike-timing is not incorporated in the learning rule, the learning is not exclusively rate-based. Namely, since the output spikes are filtered over time, the total contribution of one output spike is still timing-based. This timing has to be learned.

### 2.5 Environment: Pong

In this work, we investigated spatiotemporal ballistic control. We chose to adapt the game of *Pong*, and used it as a toy problem. The *Pong* game is an Atari game in which two players each control a paddle on opposite sides of the playing field. A ball moves from one side to another, and can bounce on the top and bottom walls. The goal for the players is to return the ball to the other player by hitting it with the paddle, such that the other player is not able to return it. A point is scored when one player misses the ball, and the game resets to an initial state.

For the purpose of evaluating the usefulness of our algorithm, the adapted version of the game needed to adhere to several requirements:

- The game and system characteristics should be spatiotemporal
- It should be possible to gradually change the game's difficulty for benchmarking
- The environment needs to be simple
- Performance needs to be measured straightforwardly
- It should be possible to generalize the model to situations or tasks by using additional constraints.

**Figure 4:**
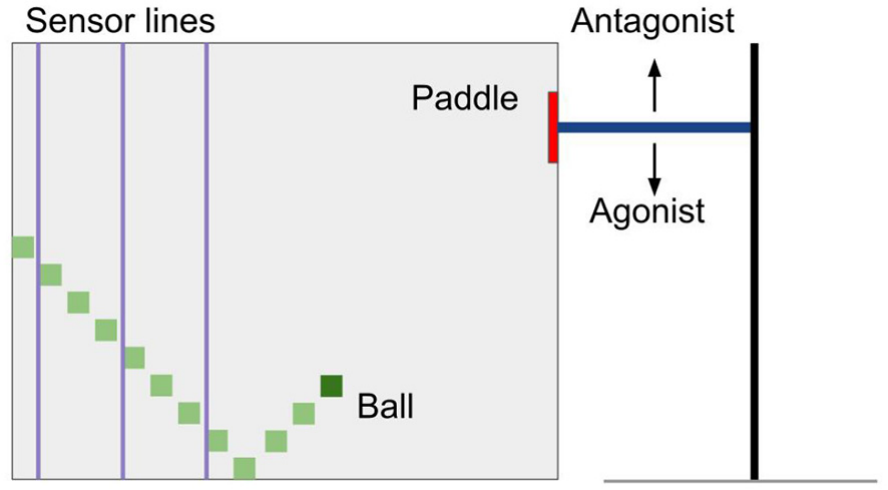
Pong game environment. The ball is shot from the left side at an angle ϕ. The sensor lines detect the ball's position (*x*_*b*_, *y*_*b*_). When the ball passes the last sensor line, the input neurons start firing. Output twitches move the paddle. In this example, we have three sensor lines. This is the minimum number of sensors needed to differentiate between states when the ball can bounce once.

In our adapted game of *Pong* there is one agent that controls the paddle. In every trial, the ball is shot from one side to the side of the agent's paddle under a pre-specified angle and always with the same speed. The goal for the agent is to intercept the ball. The agent's paddle is randomly initialized at the start of every trial. Inspired by the cricket batsman, who observes the ball at three different moments in time before he swings the bat (Land and McLeod, 2000), we chose to detect the ball using models of three equally separated ToF sensor lines. The sensor lines crossed from the top to the bottom of the environment. The position of these ToF lines was predetermined. When the ball passes a ToF's receptive field, the sensor detects the ball and emits a signal proportional to the height of the crossing ball. When the ball has crosses the third sensor, the sensor-crossing position data, as well as the paddle position data are converted into spiking signals by Gaussian encoding. Consequently, the input neurons start spiking stochastically, and the spikes are processed by the rest of the network. The output motor units are driving a paddle controller. One half of the neurons push the paddle up, the other half pushes the paddle down. A visualization of the environment can be seen in Figure 4. After a hit or a miss, the distance from the middle of the paddle to the ball is determined and used as global error. Weight updates are performed before the game is reset.

## 3 Results

### 3.1 Environment adaptation

An adapted variant of the game of *Pong* is designed such that the difficulty of the game can be set by tuning several game-parameter settings. In order to find usable hyperparameter settings for SIF-DFA, a pilot experiment was performed. The environment (2,000 × 1,600 pixels) contains three equally spaced virtual ToF sensors on 5, 25, and 45% of the distance from the shooting side to the paddle side. The paddle height is 15.6% of the environment height. The ball has an absolute velocity of 1 pixel per timestep, and is shot with a gradient between [−0.5, 0.5], which corresponds to an angle between [−26.6°, 26.6°]. We train three-layer TwNNs's, with either 100 or 200 input neurons, 50 hidden neurons, and 100 output neurons. For the hidden and output neurons, a neuron threshold of 20 *a*.*u*. is chosen. This corresponds to 10 or 20% of the maximum input value for the hidden units per timestep and 40% of the maximum input value for the output units per timestep. The two time constants for each motor unit α and β are drawn from a uniform distribution *U*(50, 200), which translates to sampling from a decay factor range between approximately 0.98 and 0.995. Similar to the feedforward weights, in order to make the feedback weights more easily implementable in electronics, ultimately, they are drawn from a uniform distribution *U*(−1, 1).

### 3.2 Baseline SIF-DFA

Firstly, we investigated the simplest version of our algorithm, which counts as the baseline algorithm. We fully connected an input, hidden, and output layer. We ran parameter sweeps for 200.000 trials per parameter combination. The parameter values can be seen in Table 1. The motor unit model amplitudes are drawn from a uniform distribution *U*(1, *MaxAmp*), as this is one of the most uninformed distributions to draw from. In order to ensure weight stability and promote diversification of output signals, we clipped the weights between [−1, 1] (Elsayed et al., 2024). The performance of the system is determined by averaging the hit rate of the last 10000 trials, as we can visually observe that in most cases, training has stabilized by then. For the next part of the result section, to prevent sections of text populated with statistical test results, we have grouped the tests in Table A1 in Appendix.

**Table 1:** Parameters and values of the initial parameter sweep.

Parameter	Values
α	0.1, 0.01, 0.001, 0.0001, 0.00001
ζ	2, 5
#SensoryNeurons	25, 50
Receptivity	25, 50
Steepness	100, 1000
Max. amplitude [a.u.]	25, 100

α represents the learning rate. ζ represents the excitability of the input neurons. *#SensoryNeurons* represents the number of neurons per ToF sensor, as well as paddle position sensor. *Receptivity* represents the width of the Gaussian receptive field, also *c* in Equation 1. The *Steepness* parameter is a scaling term for the accumulated activities *a*_1_ and *a*_2_ such that they lie closer to the learning regime of the Sigmoidal curve, as shown in Equation 13. *MaxAmp* represents the maximum amplitude for the motor unit model.

To assess the trained model's performance, we define a cutoff point for either good or bad interception rates. We decided an acceptable interception rate is an interception rate of 85%, as we think 80% is playable, and 90% is very good. As a result, everything below 85% interception rate is categorized as bad, whereas interception rates of 85% or higher are categorized as good.

**Figure 5:**
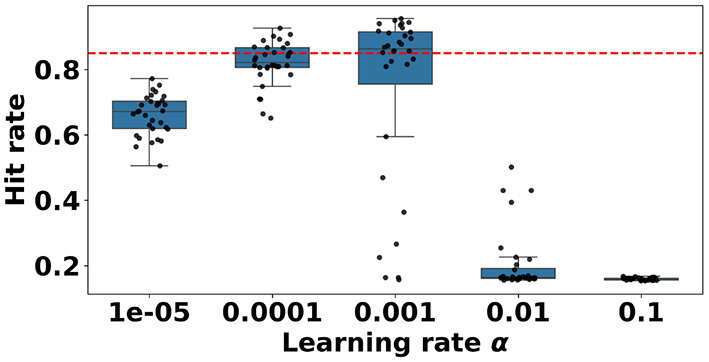
Boxplot showing the distributions of hit rates per learning rate α, resulting from a hyperparameter sweep performed with the baseline learning algorithm. The red dotted line at 0.85 indicates the cutoff between acceptable performance and unacceptable performance. Learning with learning rates of α = 1*e*−05, 0.01, and 0.1 does not achieve a single trial with a good hit rate. Using a learning rate of α = 0.001, almost all trials reached an acceptable performance.

The performance results of training a model for each parameter combination, together with the cutoff point, can be seen in Figure 5. Out of 160 models with different parameter combinations, 31, or 19.4% models learned to intercept the ball above an 85% interception rate. We can see that the learning rate has a significant influence on the ability of the model to learn (Table A1). The occurrences of a learning rate of both 0.001 and 0.0001 in the best-performing trials are above the expected counts. The performance for other parameter value combinations does not differ significantly in the good trials. We can see that learning rates of α = 1*e*−05, 0.01, and 0.1 do not achieve a single trail with a good hit rate. The performances of models trained with a learning rate of α = 0.001 and 0.0001 do pass the 0.85 threshold line. All parameter combination performances can be seen in Supplementary Figure S1.

### 3.3 Variant: stabilizing the learning process

**Figure 6:**
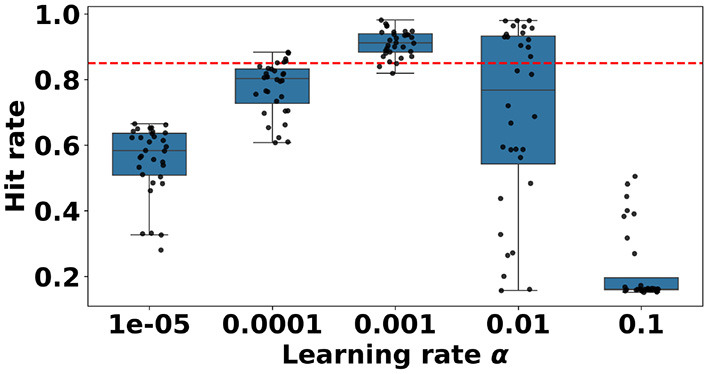
Boxplot showing the distributions of hit rates per learning rate α, resulting from a hyperparameter sweep performed with the SIF-LFA learning rule. The red dotted line at 0.85 indicates the cutoff between acceptable performance and unacceptable performance. Learning with learning rates of 1*e*−05, and 0.1 does not achieve a single model with a good hit rate. Almost all models with a learning rate of 0.001 achieve a good hit rate.

Individual output neurons can significantly differ in their spiking activity. This affects the speed with which they learn. In nature, a linear increase in stimulus does not necessarily result in a linearly increased intensity of perception (Demarchi et al., 2025; Stevens, 1957; Fechner, 1860; Weber, 1834). As such, we use the “perceived error” rather than the absolute error to adjust the weights. In order to make learning more stable, at the end of each trial, we apply a logarithmic transformation to each neuron's cumulative output activity. We name this variant SIF-LFA. The logarithmic transformation can be cheaply realized by a transistor in its subthreshold regime. Rather than *e* = *Ec*_*n*_*A*_*n*_, the individual error now defined as *e*_*log*_ = *E* ln(*c*_*n*_+1)*A*_*n*_. Maintaining the same cutoff point of 0.85, 49 out of 160 (30.6%) of observations reach a good interception rate. This interception rate is significantly better than the interception rate of the baseline approach (Table A1). Similar to the baseline approach, the learning rate has a significant effect on the good accuracies (Table A1). Training runs with learning rates of 0.001 and 0.01 result in more acceptable interception rates than the expected, average count. Models with a maximum amplitude of 25 pixels perform better than models with a maximum amplitude of 100 pixels (Table A1). We visualize the learning rate dependency in Figure 6. A learning rate of α = 0.001 produces models that mostly surpass the 85% threshold. A more detailed view of the parameter sweep can be seen in Supplementary Figure S2.

### 3.4 Variant: avoiding summation circuits

Counting neuron spikes and summing activities requires analog-to-digital converters, adders, and accumulator circuits. Such circuits are bulky and energy intensive as they need a constant power supply. Instead of using summations, in this variant on the learning rule, we use leaky integrators to keep track of neuron activity. As such, the learning signals will not be based on the precise activity histories, but proxies thereof. In electronics, the decay can be realized by slowly discharging capacitors or volatile memristors with relatively high time constants (Wang et al., 2017; Ju and Kim, 2024; Griffin and Redmond, 2024; Ricci et al., 2022) as not to lose information too fast. In the literature, these decay factors are often called eligibility traces. As such, we call this method SIF-EFA.

**Figure 7:**
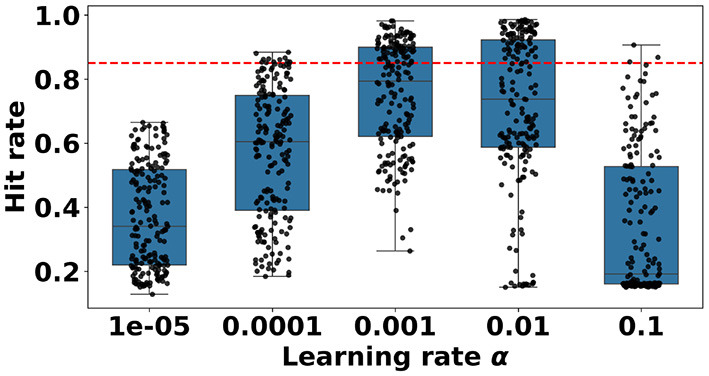
Boxplot showing the distributions of hit rates per learning rate α, resulting from a hyperparameter sweep performed with the SIF-EFA learning rule. The red dotted line at 0.85 indicates the cutoff between acceptable performance and unacceptable performance. Learning with a learning rate of 1*e*−05 does not produce a single model with a good hit rate. Learning with a learning rate of 0.1 produced only a few models with a good hit rate. Note that compared to SIF-LFA, the only difference is the parameter τ. Learning with this parameter produces many bad models, although the concentration of good models still seems to be high at learning rates 0.001 and 0.01.

We performed a parameter sweep over the same parameters as in Table 1, with the addition of parameter τ∈{0.9, 0.95, 0.99, 0.995, 0.999}, representing the leak factor of the count per timestep. New definitions for the activation and output spike history become: ${\text{a}}_{1}^{\prime }(t+1)=({\text{a}}_{1}^{\prime }(t)+W_{1}\text{x}(t+1)))\tau$; ${\text{a}}_{2}^{\prime }\left(t+1\right)=\left({\text{a}}_{2}^{\prime }\left(t\right)+W_{2}{\text{h}}_{1}\left(t+1\right)\right)\tau$; $c_{n}^{\prime }\left(t+1\right)=\left(c_{n}^{\prime }\left(t\right)+s_{out}\left(t+1\right)\right)\tau$. Identical to the learning stabilization variant, the summed state variables are log-normalized before updating the weights. Out of 800 models with different parameter combinations, 122 models (15.3%) achieve a good performance. This is significantly lower than the log-normalized performance without decay (Table A1). Again, the learning rate has a significant effect on learning (Table A1). Learning rates of 0.001 and 0.01 result in better performance than the expected value. A maximum amplitude of 25 pixels is significantly better than a maximum amplitude of 100 pixels (Table A1). A steepness value of 100 also results in significantly better performance than a steepness value of 1,000 (Table A1). Eligibility trace's decay rates also have a significant effect on learning (Table A1). The lower the eligibility trace value, the lower the number of good trials seems to be, as can also be seen in Supplementary Figure S3. In order to compare which decay rates are significantly worse than no decay rate, we perform a χ^2^ test on the entire data set for each decay rate. We use the Bonferroni correction to compensate for comparing the same variable to multiple others. The significance level is α = 0.05/5 = 0.01. We conclude that learning with a τ of 0.999 results in a higher, although not significantly higher, number of good trials than learning without decay rate (51 vs. 49). Learning with a τ of 0.995 is not significantly worse than learning without decay rate (Table A1). And finally decay rates of 0.99 and lower are significantly worse than no decay (Table A1). The learning rate dependency is visualized in Figure 7.

To see whether the hit rate is consistent for different levels of difficulty, we performed a parameter sweep over the maximum ball angle ϕ_*max*_. From the SIF-EFA experiment, we took the best run where τ = 1, the best run where τ = 0.999, and the best run where τ = 0.995. The best hyperparameter combinations can be seen in Table 2.

**Table 2:** Best hyperparameter combination per τ value.

τ	α	ζ	#SensoryNeurons	Receptivity	Steepness	MaxAmp	Hit rate
0.995	0.01	2	50	50	100	25	0.986
1.0	0.001	2	50	50	1000	25	0.982
0.999	0.01	2	25	50	1000	25	0.984

**Figure 8:**
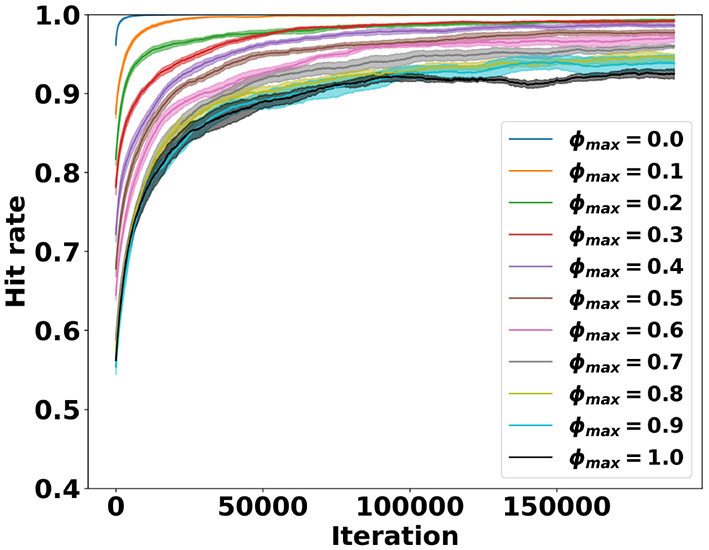
Results of the angle sweep using the model with the hyperparameters that resulted in the best run where τ = 0.995. The numbers in the legend represent the maximum gradient of the ball leaving the starting position. A gradient of 1 is an angle of 45°. We can see that increasing the maximum ball angle, and thus the difficulty, decreases learning ability. Please note that the y-axis starts at 0.4.

Copying the corresponding hyperparameter values, we ran each combination 5-fold, for a maximum initial ball gradient of *g*∈0, 0.1, 0.2, ..., 1.0. We then computed the average and standard error. The best parameter combination in the SIF-EFA parameter sweep is shown as entry for τ = 0.995 in Table 2. We show learning curves of an angle sweep with the same hyperparameters in Figure 8. The other two best-run angle sweeps were very similar in their outcome and can be found in Supplementary material S2. We can see that the model performances drop with an increase in angle. However, they are still able to play the game well at a gradient of 1, or an angle of 45°, which is the typical maximum angle for the game of pong.

### 3.5 Threshold sensitivity analysis

**Figure 9:**
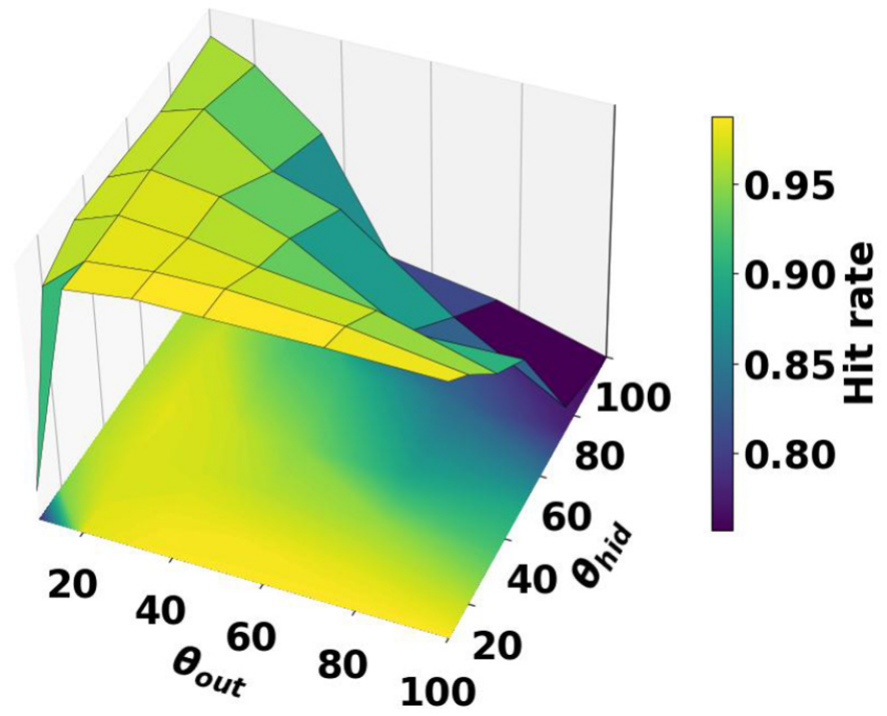
Results of the threshold sensitivity analysis. The weights and hyper parameters of the best SIF-EFA model are used to perform threshold sensitivity analysis. The best combination of thresholds seems to be a relatively low value for the hidden neurons and a relatively high value for the output neurons. This can potentially be explained by the number of inputs per layer, i.e., the number of inputs to the hidden layer is four times as high as the number of inputs on the output layer.

The neuron thresholds are of importance in spiking neural networks, especially when activities are not normalized. We analyzed the sensitivity to the threshold in training by training networks with different combinations of hidden neuron thresholds Θ_*hid*_ and output neuron thresholds Θ_*out*_. Where Θ_*hid*_, Θ_*out*_∈{10, 20, 35, 50, 75, 100}. For the purpose of this analysis, we utilize the parameter and hyperparameters of the SIF-EFA model that achieved the highest hit rate after the parameter sweep, i.e., the model corresponding to the hyperparameters where τ = 0.995 in Table 2. Each model, defined by a specific threshold-combination configuration, was trained 5 separate times using different initializations for weights and time constants. The averages of the five runs can be seen in Figure 9. A wide range of thresholds seemed to be viable, however, the top-performing models have a relatively low hidden neuron threshold. For a tabulated view, see Table A2 in Appendix.

### 3.6 Noise resistance analysis

**Figure 10:**
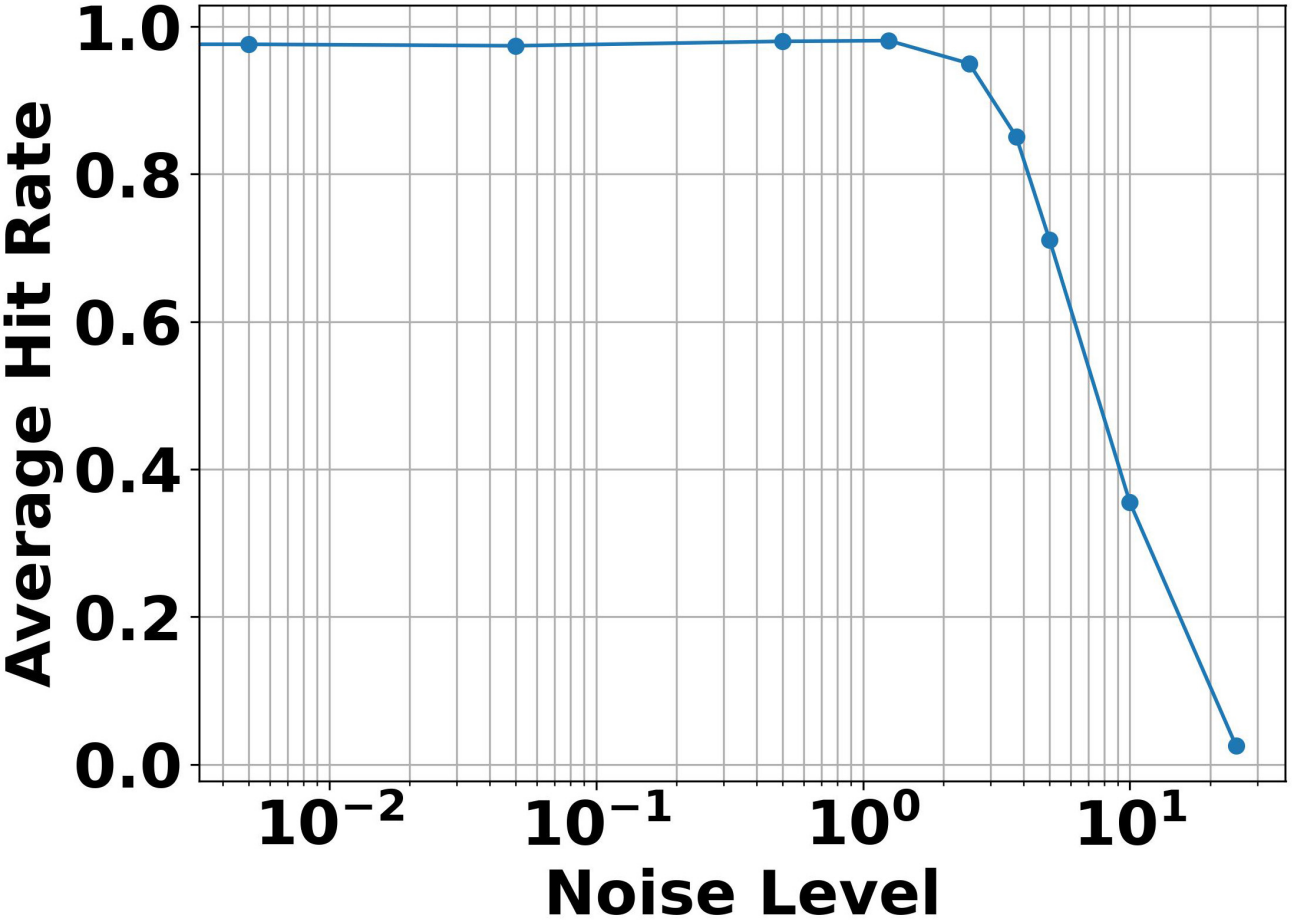
Results of the noise-resistance analysis. The performance starts to drop after a uniform noise of *N*~*U*(−1.25, +1.25), with 1.25% being the percentage of noise compared to the neuron thresholds.

During inference, the system needs to be noise-resistant. If the system is too sensitive to noise, each external perturbation in the neurons can lead to undesired actions in the output. To test the noise-resistance of the TwNN, we added different levels of uniform noise *N*~*U*(−*noiselevel*, +*noiselevel*) to the membrane potentials of the neurons at every timestep. Again, we used the parameters and hyperparameters of the best-performing SIF-EFA model. The noise levels are expressed as the percentage of the neuron thresholds. The model is tested 1,000 times per noise level. The results are shown in Figure 10. We can see that for a low noise level, the system is very robust. It starts to drop off after a noise level of 1.25%.

## 4 Discussion

We aimed to train a neuromuscular-inspired system that could learn to perform ballistic control tasks. The system is also made in anticipation of electronic implementation.

### 4.1 Neuromuscular inspiration

We implemented a spiking neural network with a muscle-inspired output by filtering the network's output spikes with second-order low-pass filters. Although the system was able to control the paddle well after training, there exist multiple optimal policies for playing the game of Pong. One more optimal policy is to stop the paddle, or to have a very low velocity when reaching the correct position to intercept the ball. This allows for repositioning faster after the ball is intercepted. No optimization to accommodate for this policy has been performed. Another improvement in learning for a better policy is to add a term that rewards the minimization of energy expenditure in controlling the paddle. When observing the agonist and antagonist activation in the simulations, we can see that they co-contract, as no penalty has been set on co-contraction in training. Although from an energy standpoint this would be beneficial, the question remains whether no co-contraction is preferable. Nature might utilize this seemingly inefficient mechanism to optimize movement or posture (Latash, 2018). Another efficiency that has evolved in nature is the distribution of motor neuron fibers. In some human muscles, small slow-twitch fibers outnumber big fast-twitch fibers (Gollnick et al., 1974), while in other muscles it is the other way around (Staron et al., 2000). It would be interesting to investigate the effect of different distributions of filter time constants and amplitudes on learning.

### 4.2 Future Implementation in electronics

One of the aims of the work was to anticipate electronic implementation. We will share our vision of possible solutions for every part of the pipeline.

Sensor system: In the encoding stage of our pipeline, Gaussians are used to convert the sensed ball position to a strip of SNN inputs. As mentioned in Section 2, different Gaussian-like function generator circuits exist. They project the input values onto a Gaussian function with programmable amplitude, mean, and variance. The output value of our Gaussian encoder is fed into a circuit which probabilistically spikes with a frequency of $\frac{value}{threshold}$. Several solutions exist with op-amps or in MOSFET to realize a transfer function with an inverted-U shape. A smooth inverted-U can be made as the difference of two sigmoidal (satiating) responses. Neural system: The processing stage of the pipeline requires spiking neuron models, tunable weights, and memory for weight updates. Many SNN integrated circuits are made (Basu et al., 2022), where the leak rate is often tunable. Setting the leak rate at or very close to zero, these can be used for our spiking neural models. Furthermore, tunable weights could be realized using a cascade of memristors. Every spiking neuron also needs a local memory, which can be used for weight updates. A cheap option would be to use a volatile memristor or a capacitor with high time constants.

Output twitches: The third part of the pipeline is the output of the network, filtering the spike signal into a smooth output. As the filters are not trainable, randomly selected second-order RC circuits with a randomly drawn amplitude can be connected to the spiking units of the processing stage. A voltage-to-current converter needs to convert the output voltage of the last RC filter into a current. Using an adder, twitch-induced currents can now be added up. The current adder can then be connected to a DC-motor where the input current is directly proportional to speed.

An alternative to using the sum of the output as driving voltage for the paddle is by using an ensemble of dielectric elastomer artificial muscles to drive the paddle (Guo et al., 2021) in a physical realization. This approach currently works only with high voltages and relatively low speeds, so it can currently only be applied in environments that do not require large movements. However, research in contractile actuators currently enjoys an increased interest Molla et al. (2026), such that time is ripe to anticipate with research on control mechanisms for (pools) of non-linear actuators. Feedback processing: The last stage of the pipeline is the learning stage. Existing neural-network models contain weights having many possible values with high precision. In principle, we used double precision (64 bits). More coarse representations of floating point are possible. Single precision will be precise enough for the gradient descent, with lower precisions leading to training problems. However, after training, the weight values can be binarized (Courbariaux et al., 2015). For memristive systems, the challenge is to realize training where weight values are a limited list of stable electric resistances. A device achieving an impressive of 20 stable resistances was discovered (Wu et al., 2019), but this is still not suitable for training mechanisms that rely on small deltas on the gradient. Such results are however hopeful and useful for realizing a sufficient diversity in output states of a neuron.

In future work, it is useful to investigate the performance as a function of weight precision and/or weight stability for the task used here. A more fundamental issue concerns the presence of bipolar weights in artificial neural networks. In the biological neural network, inhibition (negative weights) is only realized via an inhibitory interneuron. This is akin to having an inverter in electronics. However, both in the biological and in the electronic realization of a neural network, sign-flipping of a weight during training poses a fundamental problem. In the case of a realization with analog electronics, in analog electronics a bipolar DC system and bipolar amplifiers would be required. A more energy-efficient system would use only unipolar weights, only relying on an inverter and a switching solution for the inversion sign bit. Some solutions effectively assume a differential crossbar, one lead representing the positive and another representing the negative weights.

### 4.3 Advantages of twitch-based movement control in hardware

Besides the biological plausibility, the second-order overdamped impulse response may have another advantage, in preventing wear and tear at the high-power output transistor stage and the electromechanical actuator. Fast transient control voltages generally lead to more heat in transistor junctions (Mortenson, 1957) and power MOSFETs in particular (Hosseinzadehlish, 2025), potentially reducing the component life time. In the actuator, the situation is even worse: Fast transient control signals lead to inductive currents, movement overshoot, resonance and recovery curves, mechanically stressing the apparatus, its bearings, etc. (Lu et al., 2022). Due to the smoothness of the overdamped impulse response, these damaging voltage spikes are effectively low-pass filtered without requiring a separate output filter stage, avoiding stepwise or spiking transients on the final control output.

### 4.4 Neuron model

In this work, we utilized simple integrate and fire neurons. These models lie closer to feedforward ANNs than complex neuron models do, and can be more compatible with the DFA learning rule. For the Pong task, there is enough non-linearity in the thresholding and the output to solve the task using IF neurons. For more complex tasks however, the limited amount of non-linearity may pose problems. In future research, different neuron models that enhance non-linearity can be investigated.

### 4.5 Direct feedback alignment

Although DFA does not require a processor to calculate gradients flowing backwards, it does require an extra circuit that serves as a feedback matrix. An advantage is that feedback matrices can be copied and utilized in another system as long as the shape of the feedback matrix is correct. Depending on how the feedback matrices are produced, it is also possible to use a subset of a larger matrix, making larger matrices universal for different systems.

### 4.6 Generalizability

In the adapted version of Pong, several game parameters can be tuned. We have seen that a good combination of hyperparameters generalizes well when the maximum ball angle is increased, and thus the difficulty is increased. Examples of other game parameters that can be tuned are the ball's velocity, the dimensions of the environment, the positions of the ToF sensors, and the size of the paddle. The entire pipeline is generalizable under several conditions: no neural feedback during the task is necessary to complete the task, and a single ballistic movement is needed to complete the task. Time constants of the model have to be on the order of magnitude of the timescale of the to-be-completed task.

## 5 Conclusion

We have shown that it is possible to teach a neuromuscular-inspired system to learn to perform ballistic control. As a toy problem, an adapted version of the game of Pong is made. This allowed for predictive control. The game environment contained three object detection lines, of which the measurements were encoded into spikes using Gaussian encoding. Spiking neural networks were trained using a spiking version of direct feedback alignment, which we called SIF-DFA. SIF-DFA avoids update-locking in the neural network, and a simple error signal is used to drive learning. This method of updating prevents processor overhead that will be required for learning with backpropagation or vanilla DFA. A variant on SIF-DFA, where the sum of output spikes are logarithmically scaled (SIF-LFA), achieved significantly better performance than the baseline version. In SIF-DFA, and SIF-LFA, one subcircuit per neuron is needed to remember the history of state variables. Replacing the sum operator over time with leaky integrators (SIF-EFA), did not reduce performance significantly for high time constant leaks, while it did ease potential realizability and energy efficiency in electronics. Threshold sensitivity analysis has shown that a wide range of thresholds can be utilized, while the best combination is a relatively low hidden neuron threshold and a relatively high output neuron threshold. Finally, noise analysis during inference shows that uniform noise on the hidden and output neuron membrane potentials only starts to significantly impact performance after it reaches 1.25% of the threshold per timestep.

## Data Availability

The data generated to create the plots can be found in the Zenodo repository https://doi.org/10.5281/zenodo.17832288. The software written for this study can be found in the Zenodo repository https://doi.org/10.5281/zenodo.17832222.

## Footnotes

1
https://github.com/JordiTi/NeuromorphicPongControl

## A Statistical results

**Table A1:** Summary table of statistical tests and their results.

Variant	Interpretive result	Statistical test result
SIF-DFA	There is an effect of learning rate on learning	χ^2^(4, N=31) = 53.032, p < 0.0001
SIF-LFA	Log normalizing the outputs results in better model performance compared to the performance of the baseline approach	χ^2^(1, N=160) = 5.4, p = 0.0201
SIF-LFA	There is an effect of learning rate on learning	χ^2^(4, N=49) = 60.490, p < 0.0001
SIF-LFA	A maximum twitch amplitude of 25 results in better performance than a maximum twitch amplitude of 100	χ^2^(1, N=49) = 4.592, p = 0.0321
SIF-EFA	Eligibility traces have a negative impact on learning	χ^2^(1, N=800) = 21.530, p < 0.0001
SIF-EFA	There is an effect of learning rate on learning	χ^2^(4, N=122) = 148.656, p < 0.0001
SIF-EFA	A maximum twitch amplitude of 25 results in better performance than a maximum twitch amplitude of 100	χ^2^(1, N=122) = 10.622, p = 0.0011
SIF-EFA	A steepness value of 100 results in better performance than a steepness value of 1000	χ^2^(1, N=122) = 9,48, p = 0.0021
SIF-EFA	Eligiblity traces' decay rates have a significant effect on learning	χ^2^(4, N=122) = 74.7, p < 0.0001
SIF-EFA	A τ value of 0.999 results in a larger number of good trials than a τ of 1.	χ^2^(1, N=160) = 0.058, p = 0.8094.
SIF-EFA	A τ value of 0.995 does not result in a significantly worse performance than a τ value of 1.	χ^2^(1, N=160) = 1.910, p = 0.1670

## B Threshold analysis

**Table A2:** Table of hit rates for each combination of hidden and output neuron thresholds after 200.000 trials.

Θ_*hid*_\Θ_*out*_	10	20	35	50	75	100
**10**	0.7509	0.9796	0.9892	0.9896	0.9915	0.9908
**20**	0.9383	0.9769	0.9848	0.9863	0.9805	0.9644
**35**	0.9604	0.9774	0.9722	0.9613	0.9484	0.9157
**50**	0.9593	0.9790	0.9690	0.9442	0.8776	0.8810
**75**	0.9543	0.9687	0.9208	0.8983	0.8131	0.7456
**100**	0.9628	0.9432	0.8875	0.7702	0.7520	0.7195

Each combination is run 5-fold. Parameters of the best-performing models are used in training. The best combination of thresholds seems to be a relatively low hidden neuron threshold and a high output neuron threshold.
