# Supplementary material for: Trainable movement control using spikes and muscle-twitch dynamics
Source: Front Neurorobot. 2026 Apr 13;20:1761767. doi: 10.3389/fnbot.2026.1761767 (PMC13111196; doi:10.3389/fnbot.2026.1761767)
Supplement: Supplementary file 1 [file Data_Sheet_1.pdf]

Supplementary Material

1 PARAMETER SWEEP OVER ALL HYPERPARAMETERS

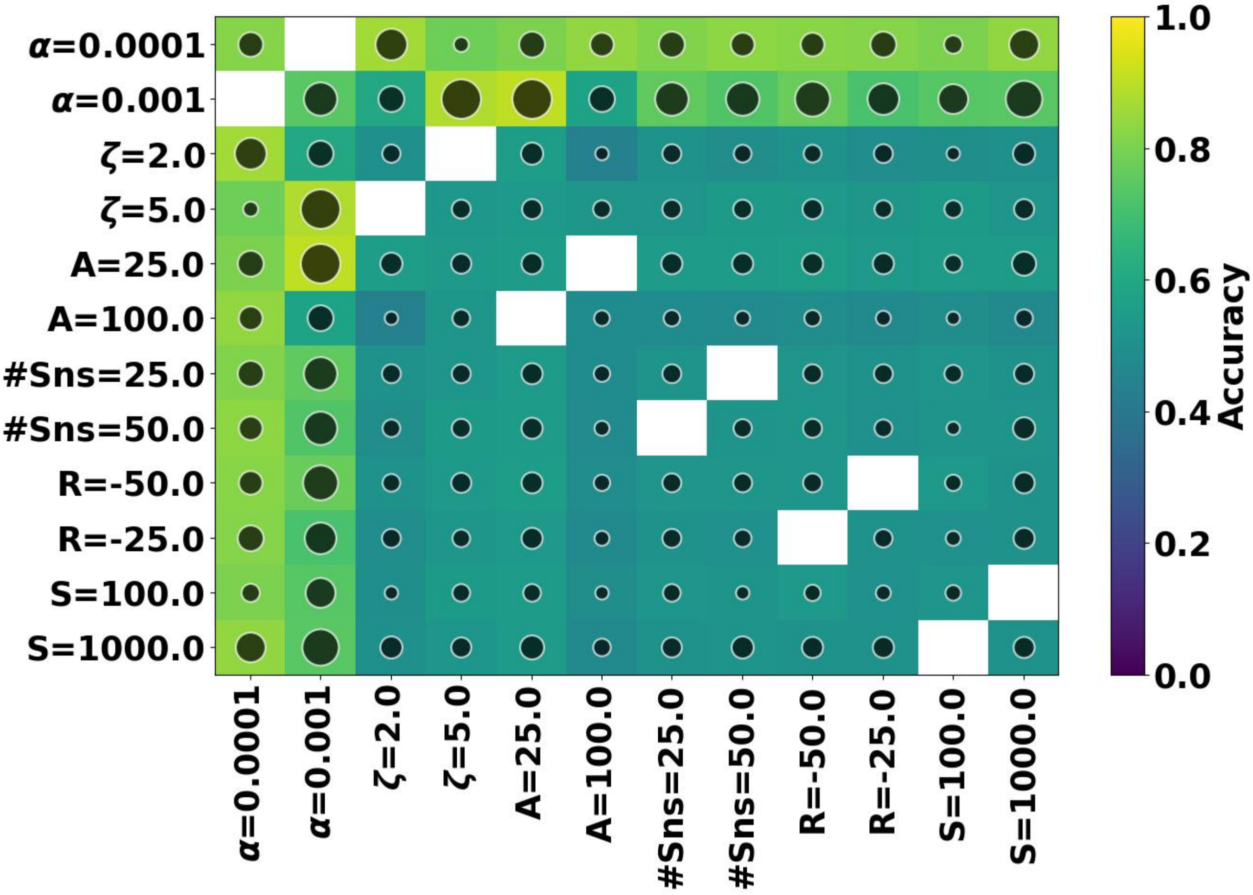

Figure S1. Accuracies, or average hit rates as a function of combinations of two parameters using the baseline learning rule. The radius of the circles in the boxes are proportional to the fraction of runs that achieved a hit rate of 0.85 or above. A circle the same diameter of the square indicates that all of the runs achieved a good hit rate. Learning rates below $\alpha = 0.0001$ and above $\alpha = 0.001$ Do not contain any trial with a good hit rate. Hence these rows and columns are not visualized. The most significant interactions besides learning rate seem to be maximum output amplitude and input threshold.

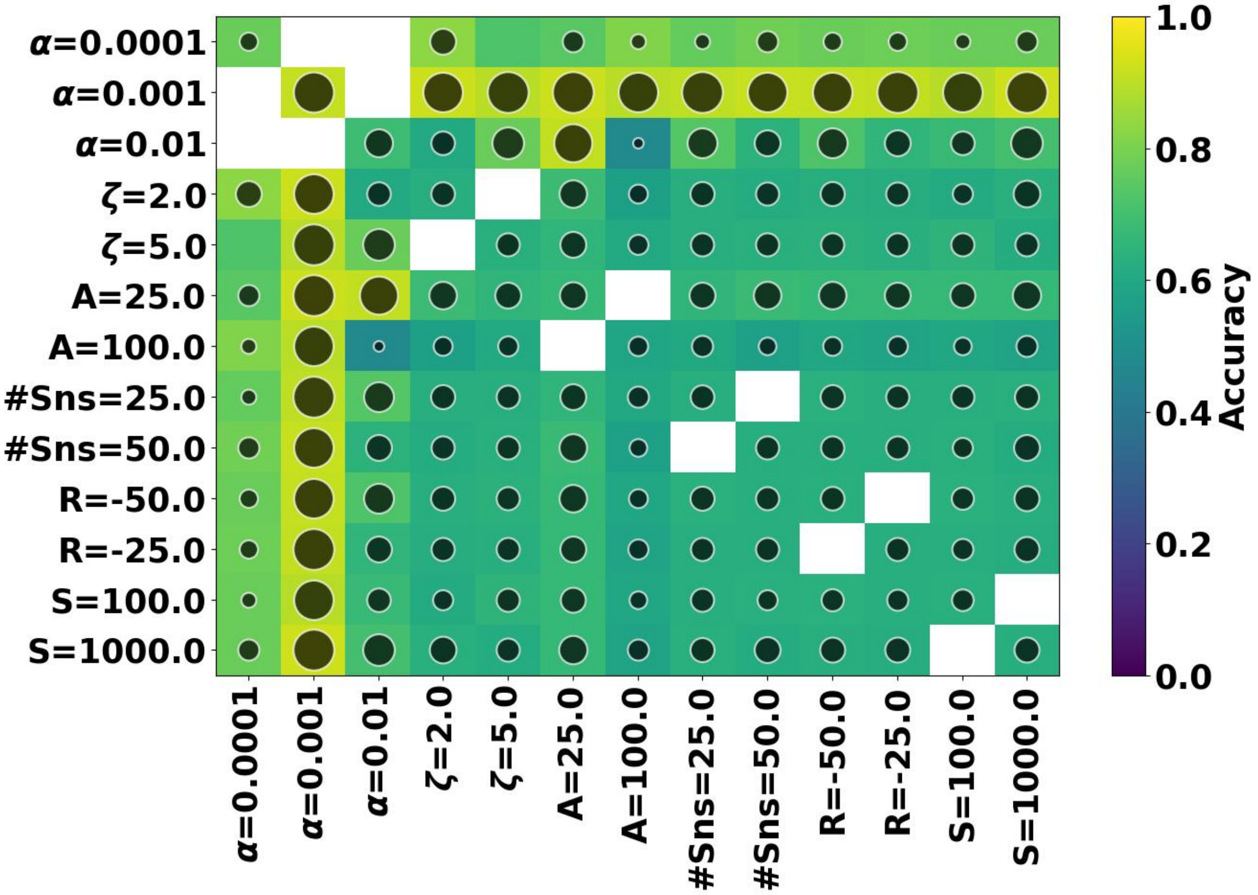

Figure S2. Accuracies, or average hit rates as a function of combinations of two parameters using the SIF-LFA rule. The radius of the circles in the boxes are proportional to the fraction of runs that achieved a hit rate of 0.85 or above. A circle the same diameter of the square indicates that all of the runs achieved a good hit rate. Learning rates below $\alpha = 0.0001$ and above $\alpha = 0.01$ Do not contain any trial with a good hit rate. Hence these rows and columns are not visualized. The most significant interactions besides learning rate seem to be maximum output amplitude and input threshold.

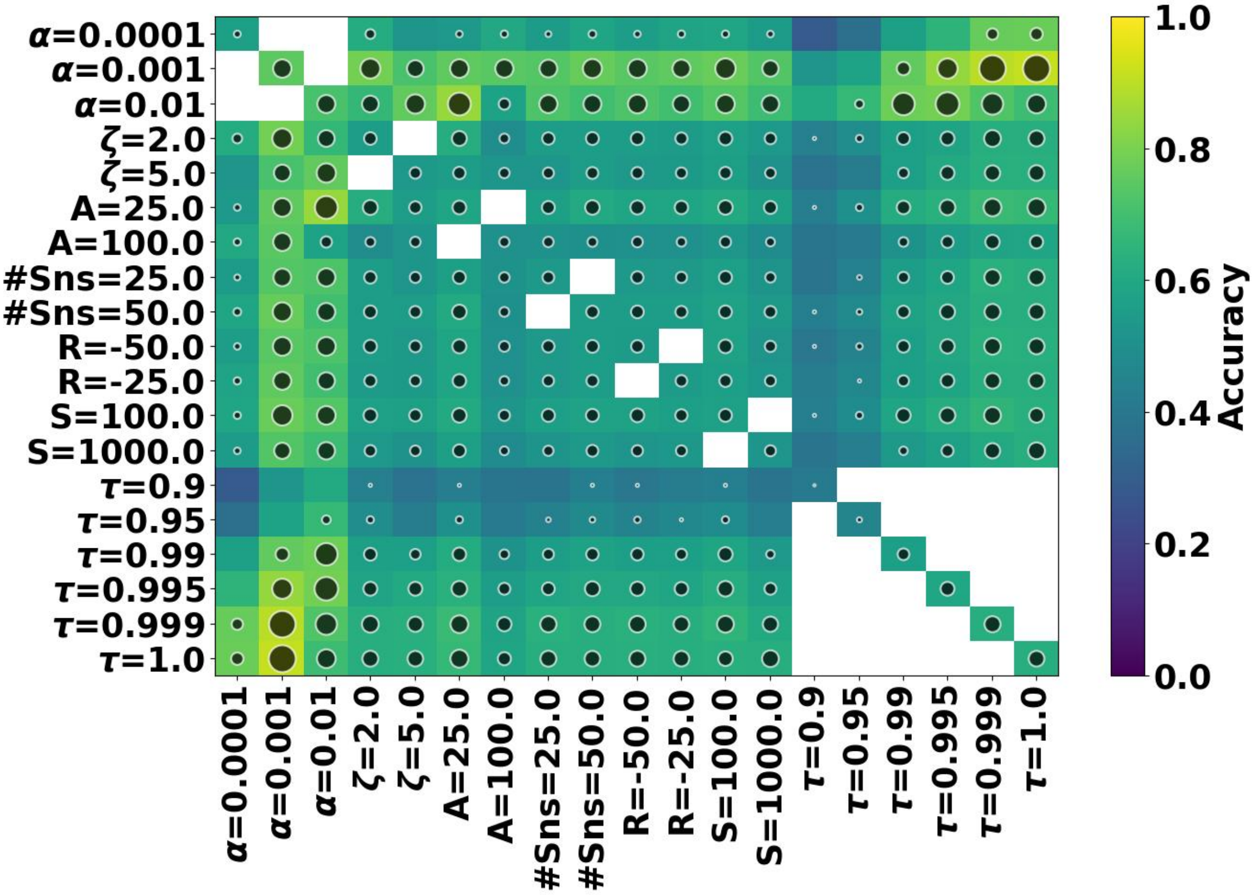

Figure S3. Accuracies, or average hit rates as a function of combinations of two parameters using the baseline learning rule. The radius of the circles in the boxes are proportional to the fraction of runs that achieved a hit rate of 0.85 or above. A circle the same diameter of the square indicates that all of the runs achieved a good hit rate. Learning rates below $\alpha = 0.0001$ and above $\alpha = 0.01$ Do not contain any trial with a good hit rate. Hence these rows and columns are not visualized.

2 PARAMETER SWEEP OVER INITIAL BALL ANGLES

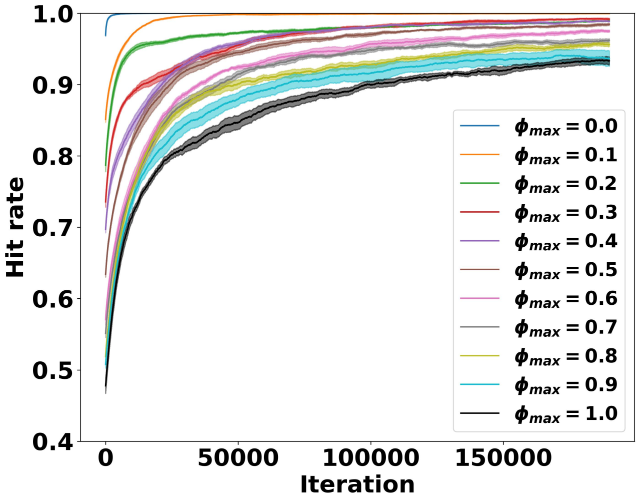

Figure S4. Parameter sweep over initial maximum ball angles using the hyperparameters of the best run with $\tau = 0.999$. A gradient of 1 indicates an angle of $45°$

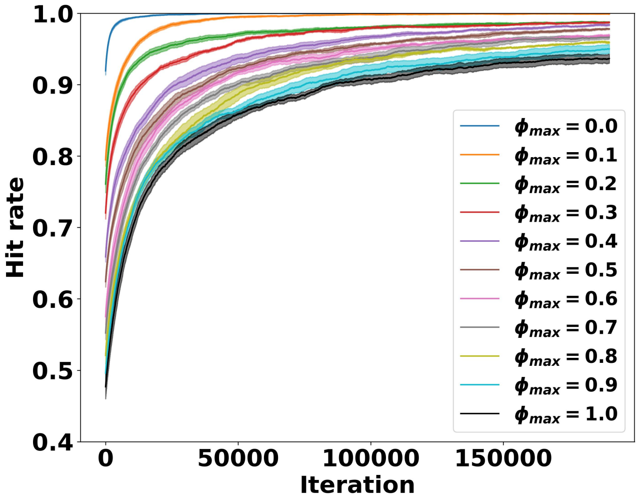

Figure S5. Parameter sweep over initial maximum ball angles using the hyperparameters of the best run with $\tau = 1$. A gradient of 1 indicates an angle of $45°$

Table S1. Results of the angle sweep using the model with the hyperparameters that resulted in the best run for $\tau = 0.995$

Maximum Angle	Average Hit Rate	Standard Error
0.0	1.000	0.000
0.1	0.999	0.000
0.2	0.993	0.002
0.3	0.992	0.002
0.4	0.986	0.003
0.5	0.977	0.005
0.6	0.971	0.007
0.7	0.960	0.003
0.8	0.945	0.008
0.9	0.938	0.014
1.0	0.925	0.009

Table S2. Results of the angle sweep using the model with the hyperparameters that resulted in the best run for $\tau = 0.999$

Maximum Angle	Average Hit Rate	Standard Error
0.000	1.000	0.000
0.100	1.000	0.000
0.200	0.989	0.001
0.300	0.992	0.002
0.400	0.989	0.001
0.500	0.984	0.002
0.600	0.975	0.002
0.700	0.962	0.003
0.800	0.956	0.004
0.900	0.936	0.015
1.000	0.933	0.008

Table S3. Results of the angle sweep using the model with the hyperparameters that resulted in the best run for $\tau = 1$.

Maximum Angle	Average Hit Rate	Standard Error
0.000	1.000	0.000
0.100	0.999	0.000
0.200	0.987	0.001
0.300	0.987	0.001
0.400	0.984	0.002
0.500	0.978	0.001
0.600	0.969	0.002
0.700	0.966	0.003
0.800	0.959	0.002
0.900	0.950	0.009
1.000	0.936	0.009
